# Supplementary material for: Dual-Functional Liposomes with Carbonic Anhydrase IX Antibody and BR2 Peptide Modification Effectively Improve Intracellular Delivery of Cantharidin to Treat Orthotopic Hepatocellular Carcinoma Mice
Source: Molecules. 2019 Sep 12;24(18):3332. doi: 10.3390/molecules24183332 (PMC6767275; doi:10.3390/molecules24183332)
Supplement: Supplementary file 1 [file molecules-24-03332-s001.pdf]

Supplementary material

Dual-functional Liposomes with Carbonic Anhydrase IX Antibody and BR2 Peptide Modification Effectively Improve Intracellular Delivery of Cantharidin to Treat Orthotopic Hepatocellular Carcinoma Mice

Xue Zhang [1,2,3], Congcong Lin [2,4], Waikei Chan [2], Kanglun Liu [2], Aiping Lu [2,5], Ge Lin [6], Rong Hu [1,3], Hongcan Shi [1,3], Hongqi Zhang [2,*] and Zhijun Yang [2,5,*]

1 Institute of Translational Medicine, Medical College, Yangzhou University, Yangzhou 225001, China; zhangxueflora@163.com (X.Z.); prhurong@163.com (R.H.); shihongcan@yzu.edu.cn (H.S.)
2 School of Chinese Medicine, Hong Kong Baptist University, Hong Kong, China; 14485680@life.hkbu.edu.hk (C.L.); nickie@hkbu.edu.hk (W.C.); liukanglun@hkbu.edu.hk (K.L.); aipinglu@hkbu.edu.hk (A.L.)
3 The Key Laboratory of Syndrome Differentiation and Treatment of Gastric Cancer of the State Administration of Traditional Chinese Medicine, Yangzhou, 225001 China;
4 Department of Medicinal Chemistry and Natural Medicine Chemistry, College of Pharmacy, Harbin Medical University, Harbin 150081, China;
5 Changshu Research Institute, Hong Kong Baptist University, Changshu Economic and Technological Development (CETD) Zone, Changshu 215505, Jiangsu Province, China;
6 School of Biomedical Sciences, Chinese University of Hong Kong, Hong Kong, China; linge@cuhk.edu.hk
* Correspondence: hqzhang@hkbu.edu.hk (H.Z.); yzhijun@hkbu.edu.hk (Z.Y.), Tel.: +852-3411-2961 (Z.Y.)

Supplementary methods

GC-MS method to analysis drug concentration

To analyze cantharidin concentration, the gas chromatography-mass spectrometry (GC-MS) was employed according to our previous study [1]. Briefly, GC-MS was performed on a Shimadzu QP-2010 instrument equipped with a Shimadzu QP 2010S mass spectrometer (Shimadzu Corporation, Tokyo, Japan). Samples were injected in splitless mode on DB-5 MS analytical column (30 m × 0.32mm ID with a film thickness of 0.25 μm film thickness, J&W Scientific, Folsom, CA). Helium was used as a carrier gas. The flow rate of gas was 1 mL/min. Injector temperature was set at 280 °C. The initial column temperature was 60°C, and then increased to 275 °C at 10 °C/min, and then to 285 °C at 20 °C/min, held for 3min. Temperature of the ion source and auxiliary temperature were 250 °C and 285 °C, respectively. Calibration curves were linear in the range of 0.1μg/ml-100 μg/ml ($r^2 \geq 0.991$). Total run time was 40 min.

In vitro drug release

Drug release behaviors of CTD from liposomes *in vitro* were performed by a dynamic dialysis technique as previously described [2]. First, we put 1 ml of liposome solution entrapped CTD or free CTD solution into a dialysis bag (MWCO 12000-16000) and tightened the both ends of the bag with cotton thread. The dialysis bag was then fully immersed in the wide mouth packer bottles containing 5 mL of PBS (pH 7.4) as release medium. Then these assemblies were put into a shaker with 50 rpm shaking at 37 ± 0.2 °C to mimic the *in vivo* conditions. At predetermined intervals, the release medium (0.2 ml) was collected for analysis and the 0.2 ml of fresh medium was added

subsequently. The samples were analyzed by GC-MS after extraction with ethyl acetate. The cumulative release rate (%) was calculated as following:

$$\text{Cumulative release rate (\%)} = \frac{c_n v_0 + \sum_{i=0}^{n-1} c_i v_i}{W} \times 100\%$$

where C_n is the CTD drug concentration in the release medium at each time point, V_0 is the total volume of the release medium, C_i and V_i is the drug concentration in the release medium at time *i* and the volume of the withdrawn medium, respectively. W is the total CTD drug content at the initial time.

Supplementary figure

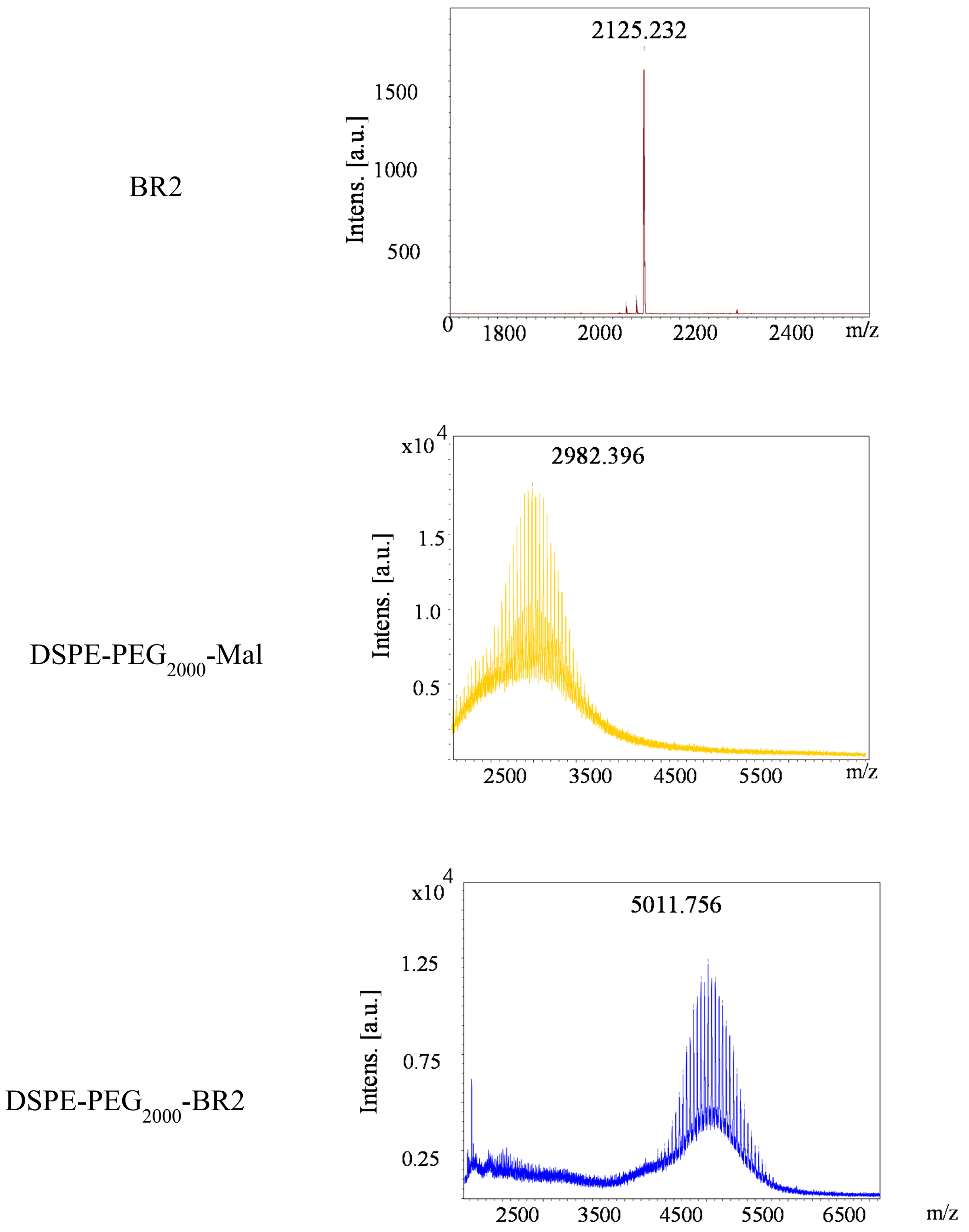

Fig. S1 BR2 cell penetrating peptide was firstly conjugated to the DSPE-PEG-Maleimide group and confirmed by MALDI-TOF/TOF mass spectra with an obvious right shift of the DSPE-PEG-BR2.

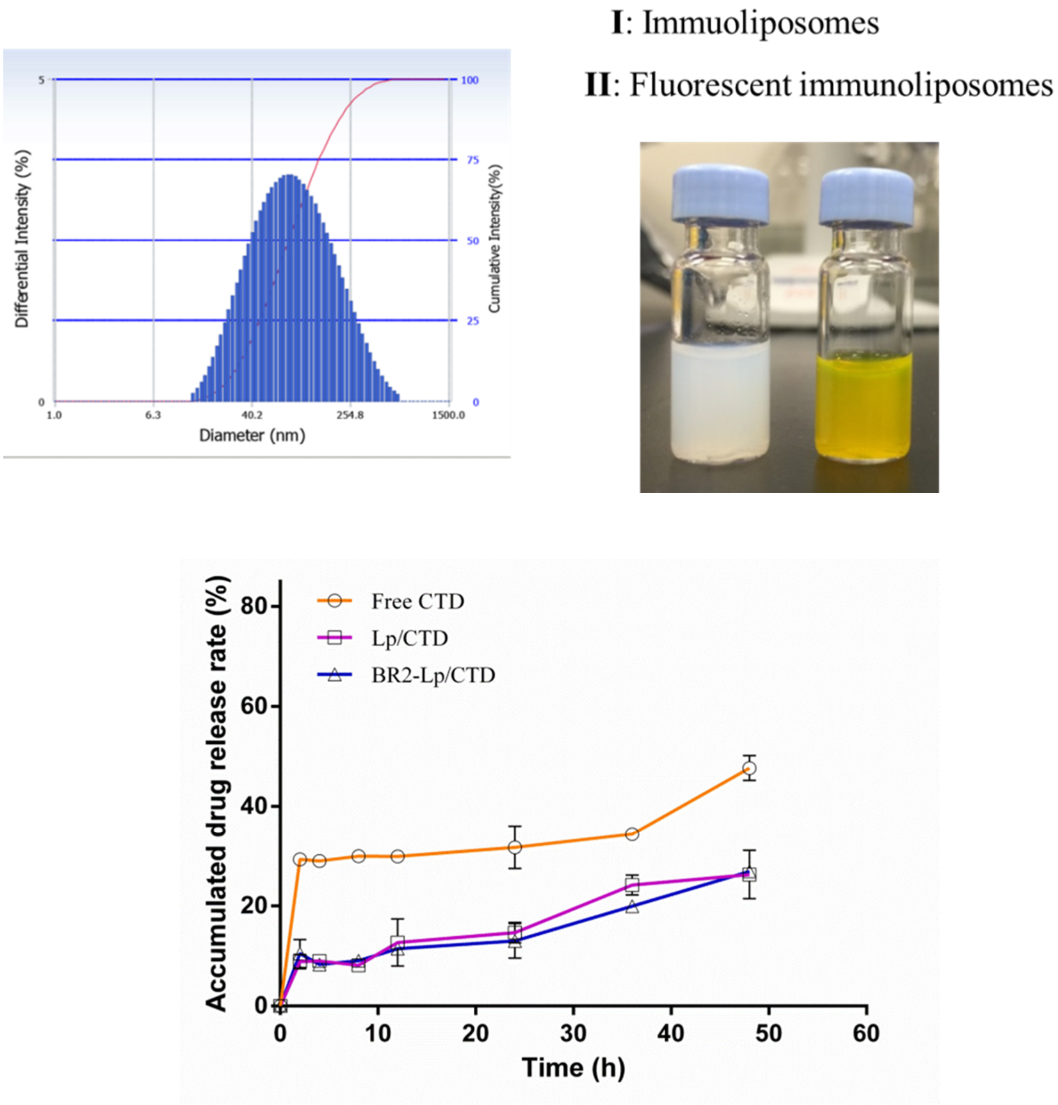

Fig. S2 The particle size and distribution of DF-Lp/CTD and the physical appearances of CTD encapsulated (I) and NBD-DPPE-labeled (II) dual-functionalized liposomes. The CTD release profile of free CTD, Lp/CTD and BR2-Lp/CTD in PBS over 48 h (n = 3, mean ± SD).

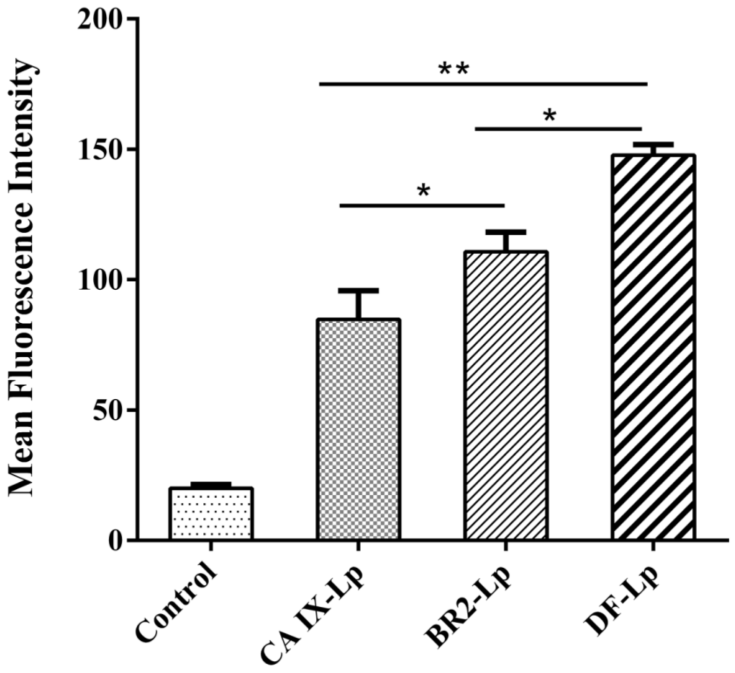

Fig. S3 The amount of cell association of different liposomes was measured by the NBD-DPPE fluorescence intensity by Image J as in the method part. One-way ANOVA analysis with Tukey's multiple comparisons were conducted between tested groups. *$P < 0.05$, **$P < 0.01$.

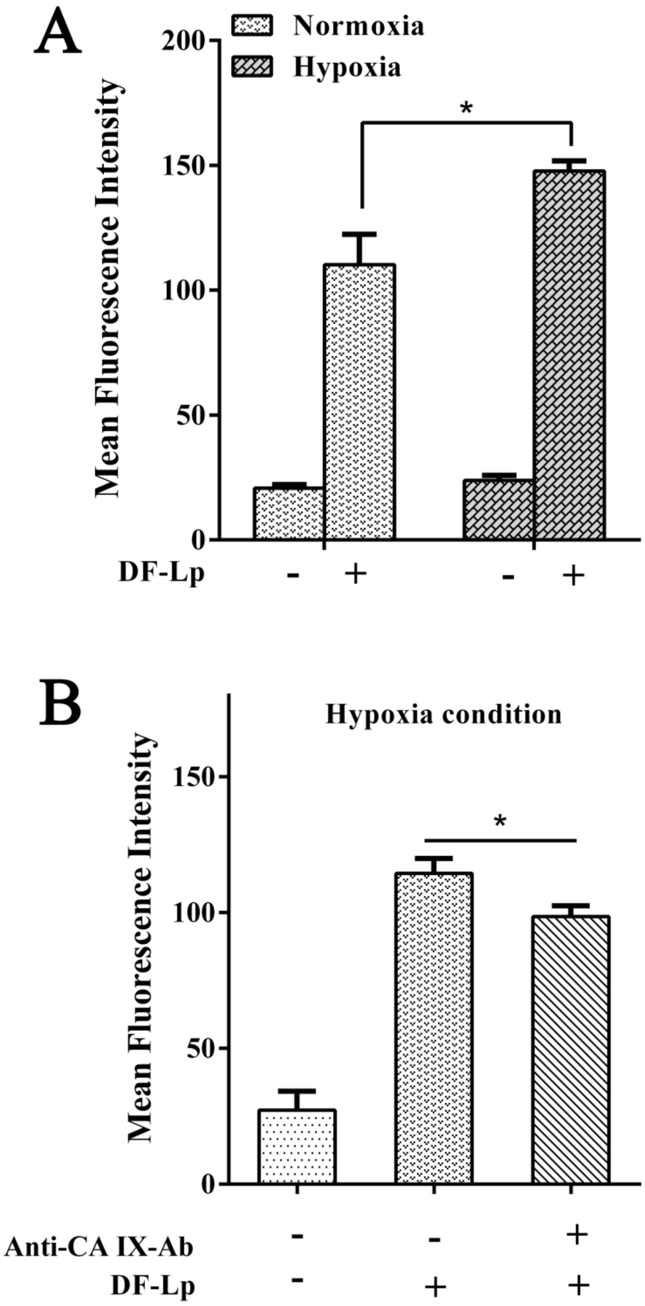

Fig. S4 (A) The NBD-DPPE fluorescence intensity after DF-Lp treatment and (B) the competition assay of CA IX antibody was analyzed with unpaired t-test to compare the significant difference between the two groups.by Image J software. *P < 0.05

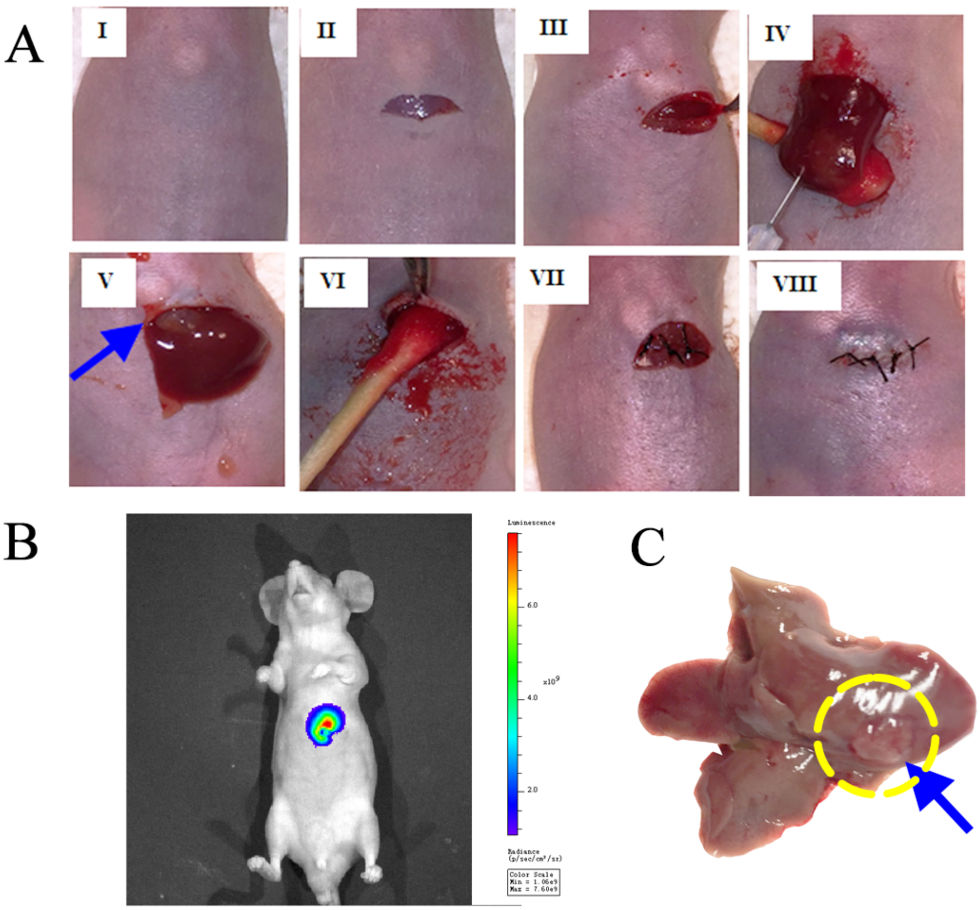

Fig S5 (A) HCC orthotopic model was developed by intrahepatic injection of HepG2-red-Fluc transfected cells with a surgical incision under general anesthesia (5% chloral hydrate 120 μl / 20 g) (I). (II) A transverse skin incision and (III) muscle layer incision on the upper abdomen under the sternum. (IV) Injection of cells slowly into the liver. (V) A transparent bleb could be seen (blue arrow). (VI) Returning the liver with a moist sterile cotton swab. (VII) Closure of muscle layer and (VIII) the skin with 5-0 suture. (B) IVIS bioluminescent image of orthotopic HCC nude mice after i.p. injection of 150 mg/kg D-luciferin. (C) Liver specimen with HCC being pointed by a blue arrow and the yellow circle.

1 Zhang X, Lin CC, Chan WK, Liu KL, Yang ZJ, Zhang HQ: Augmented Anticancer Effects of Cantharidin with Liposomal Encapsulation: In Vitro and In Vivo Evaluation. Molecules 2017, 22:1052.

2 Dang YJ, Zhu CY: Oral bioavailability of cantharidin-loaded solid lipid nanoparticles. Chin Med 2013, 8:1.
